# Enhancing Parental Motivation to Monitor African American Adolescents’ Diabetes Care: Development and Beta Test of a Brief Computer-Delivered Intervention

**DOI:** 10.2196/resprot.3220

**Published:** 2014-09-18

**Authors:** April Idalski Carcone, Deborah A Ellis, Sylvie Naar, Steven J Ondersma, Kathleen Moltz, Baseem Dekelbab, Christine LM Joseph

**Affiliations:** ^1^Prevention Research CenterDepartment of PediatricsWayne State University School of MedicineDetroit, MIUnited States; ^2^Merrill Palmer Skillman Institute and Department of Psychiatry & Behavioral NeurosciencesWayne State UniversityDetroit, MIUnited States; ^3^Children's Hospital of MichiganDepartment of EndocrinologyWayne State University School of MedicineDetroit, MIUnited States; ^4^Beaumont Children’s Hospital & St. John Providence Children’s HospitalWayne State University and Oakland UniversityDetroit, MIUnited States; ^5^Department of Public Health SciencesHenry Ford Health SystemDetroit, MIUnited States

**Keywords:** parenting, diabetes mellitus, Type 1, motivational interviewing, health behavior, intervention studies, African Americans, computer-assisted instruction

## Abstract

**Background:**

African American youth are at increased risk for poor diabetes management. Parenting behaviors such as parental monitoring are significant predictors of youth diabetes management and metabolic control, but no intervention has targeted parental monitoring of daily diabetes care.

**Objective:**

The purpose of the present study was to develop and pilot test a three-session computer-delivered intervention to enhance parental motivation to monitor African American pre-adolescents’ diabetes management.

**Methods:**

The 3 Ms (Medication, Meter, and Meals) intervention was based on the Information-Motivation-Behavioral Skills (IMB) model of health behavior change and Motivational Interviewing approaches. Five caregivers of African American youth aged 10-13 years diagnosed with type 1 diabetes for a minimum of one year (ie, the target population) reviewed the intervention and provided feedback via semi-structured interviews. Interviews were transcribed and analyzed using thematic analysis.

**Results:**

Caregivers’ responses to interview questions suggest that The 3 Ms was helpful (minimum rating was 8 out of 10) and they would recommend the program to another parent of a child with diabetes (minimum rating was 9 out of 10). Three of five reported that The 3 Ms program increased the likelihood that they would talk to their child about diabetes. Thematic analysis suggested two primary themes: caregivers found the intervention to be a useful reminder of the importance of supervising their child’s diabetes care and that it evoked a feeling of shared experience with other parents.

**Conclusions:**

The 3 Ms computer-delivered intervention for increasing parental monitoring of African-American youth with type 1 diabetes was well-received and highly rated by a small sample of representative caregivers.

**Trial Registration:**

ClinicalTrials.gov NCT01515930; http://clinicaltrials.gov/ct2/show/NCT01515930 (Archived by WebCite at http://www.webcitation.org/6Rm0vq9pn).

## Introduction

Management of type 1 diabetes (T1D) is complex, demanding, and requires daily motivation and self-control [1]. Diabetes management declines during adolescence [2-6] due to both biological [7,8] and behavioral mechanisms [2,4,5,9]. This pattern often persists into adulthood [10-12] and is associated with the development of poor metabolic control [13-15], the onset of complications [16], and increased health care costs [17]. Although African American adolescents are more likely to experience problems with diabetes management [18] and increased metabolic control [18-20], few intervention studies have focused on this group.

Maintaining parental involvement in diabetes care promotes optimal diabetes management [21-24] and metabolic control [24,25]. However, parents commonly withdraw their involvement in diabetes care as youth enter adolescence [25-28], often solely as a function of age and not youths’ capacity for autonomous self-care [21,23,29,30]. Recent studies have shown that parental monitoring of adolescents’ daily diabetes care—that is, information-seeking about their child’s diabetes care behaviors and direct supervision and oversight of those activities [31]—is a significant predictor of youth diabetes management and metabolic control [32-34]. At least one study has also found parental monitoring of adolescents’ diabetes care to be lower among non-white caregivers [35]. While other parenting behaviors and family interactions have been targeted in order to maintain appropriate diabetes care in adolescents with T1D [36-38], parental monitoring of daily diabetes care—whether in general or specifically with African-American youth—has not previously been the focus of a targeted intervention.

Motivational Interviewing (MI) [39] is a client-centered, directive method for enhancing intrinsic motivation to change problem health behaviors by exploring and resolving ambivalence. MI evokes behavior change by increasing motivation and self-efficacy through altering key decisional and self-regulatory balances by eliciting “change talk” from participants. Change talk, participants’ statements about their own desire, ability, reasons, need for, or commitment to change [40], is linked to actual behavior change [41]. MI has been widely adapted for the treatment of several health conditions including obesity [42], poor dietary practices [43], and poor diabetes management [44] in both adolescent and adult populations [45,46]. Recently, MI has been also used as a brief intervention to increase parental monitoring in populations of young children at risk for behavioral difficulties [47-49]. Additionally, MI has been successfully utilized with diverse populations in the United States and around the world with at least one meta-analysis suggesting stronger effects among minorities [46].

Despite the evidence supporting the use of MI to address poor parental monitoring of diabetes care, its integration into clinical practice is hindered by several factors. Integrating intervention programs, even brief ones, into medical practice presents significant time, financial, and logistic obstacles [50,51]. In addition, training clinicians to effectively deliver brief behavioral interventions, including MI, with a high degree of treatment fidelity is resource intensive [52-54]. On the other hand, computer-delivered interventions, once developed, hold the potential to be more easily streamlined into routine diabetes clinic visits. For example, medical assistants or other paraprofessionals who interact with patients during clinic visits could be trained to orient and log patients on to a tablet computer, which could then deliver the intervention while patients are waiting to be seen. Two recent studies have demonstrated success using laptop/tablet computers to deliver computer-based interventions in both the outpatient clinic [55] and inpatient settings [56]. In addition, integrating a behavioral intervention into routine clinical care may safeguard against common pitfalls suffered by many such interventions, including computer-delivered interventions. For example, attrition may be minimized by reducing the participation burden on the participant (ie, they do not have to make a separate trip or find time to log on to a Web-based application during a regular day) and capitalizing on a time when patients are present but unengaged (ie, they are waiting to receive their medical care).

There is growing literature supporting the use of computer-based formats to deliver brief interventions such as MI. Computer-delivered interventions offer several other advantages over traditional face-to-face interventions. The anonymity inherent in delivering an intervention by computer is associated with increased disclosure of information perceived to be sensitive [57], potentially increasing its acceptability. Computer-delivered interventions are easily replicated across persons and settings with a high degree of fidelity. Programming permits the translation of the intervention to any language and literacy level as well as individualized tailoring, a critical component of effective computerized interventions [58]. Brief, computer-delivered interventions can be widely disseminated, in this instance, delivered opportunistically (ie, during routine clinic visits) to all or most individuals, eliminating the need to screen individuals or target members of a high risk group. Broad dissemination increases an intervention’s population impact, that is, the effect of the intervention when considered across the entire population of affected individuals, even if the intervention effect is relatively small [59,60]. Although within the context of diabetes self-management, small effects are linked to significant health improvement. The Diabetes Control and Complications Trial [16] demonstrated that as little as a 10% reduction in glycated hemoglobin (HbA_1c)_ decreases the risk of complications by approximately 40%. Two recent reviews suggest computer-delivered interventions have small effects on diabetes self-management in adults with type 2 diabetes [61,62]. Together, this research provides compelling evidence to support brief, targeted interventions for diabetes.

The present study sought to develop and pilot test a brief, computer-delivered intervention targeting parental motivation to monitor pre-adolescents’ diabetes management. The intervention targeted caregivers of urban, African American pre-adolescents aged 10-13 years—youth who are beginning to assume greater responsibility for diabetes self-care and, therefore, are at increased risk for parental disengagement from diabetes care [23,63]. The newly developed intervention was a three-session, avatar-delivered, interactive program called *The 3 Ms*, which refer to the key diabetes self-care behaviors—Medication, Glucose Meter, and Meals.

## Methods

### Intervention Development

Intervention development followed the Information-Motivation-Behavioral Skills (IMB) model of health behavior change [64] and utilized approaches consistent with MI [65,66]. The IMB model posits that behavior change results from the joint function of three critical components: (1) accurate information about risk behaviors (eg, risks of letting adolescents complete diabetes care in the absence of parental monitoring) or their replacement health behaviors (eg, benefits of daily parental monitoring), (2) motivation to change behavior, and (3) behavioral skills necessary to perform the behavior (eg, self-efficacy) [64]. Thus, the goal of the intervention was to improve parental monitoring of daily diabetes care by increasing parents’ knowledge of, motivation for, and confidence in parental monitoring. The intervention was developed with the intention that it be delivered during three consecutive routine diabetes clinic appointments by clinic staff. For practices, like the study site, that adhere to the American Diabetes Association’s clinical recommendations for frequency of medical care for youth with T1D [67], the intervention sessions would be delivered at 3 to 4 month intervals.

**Figure 1:**
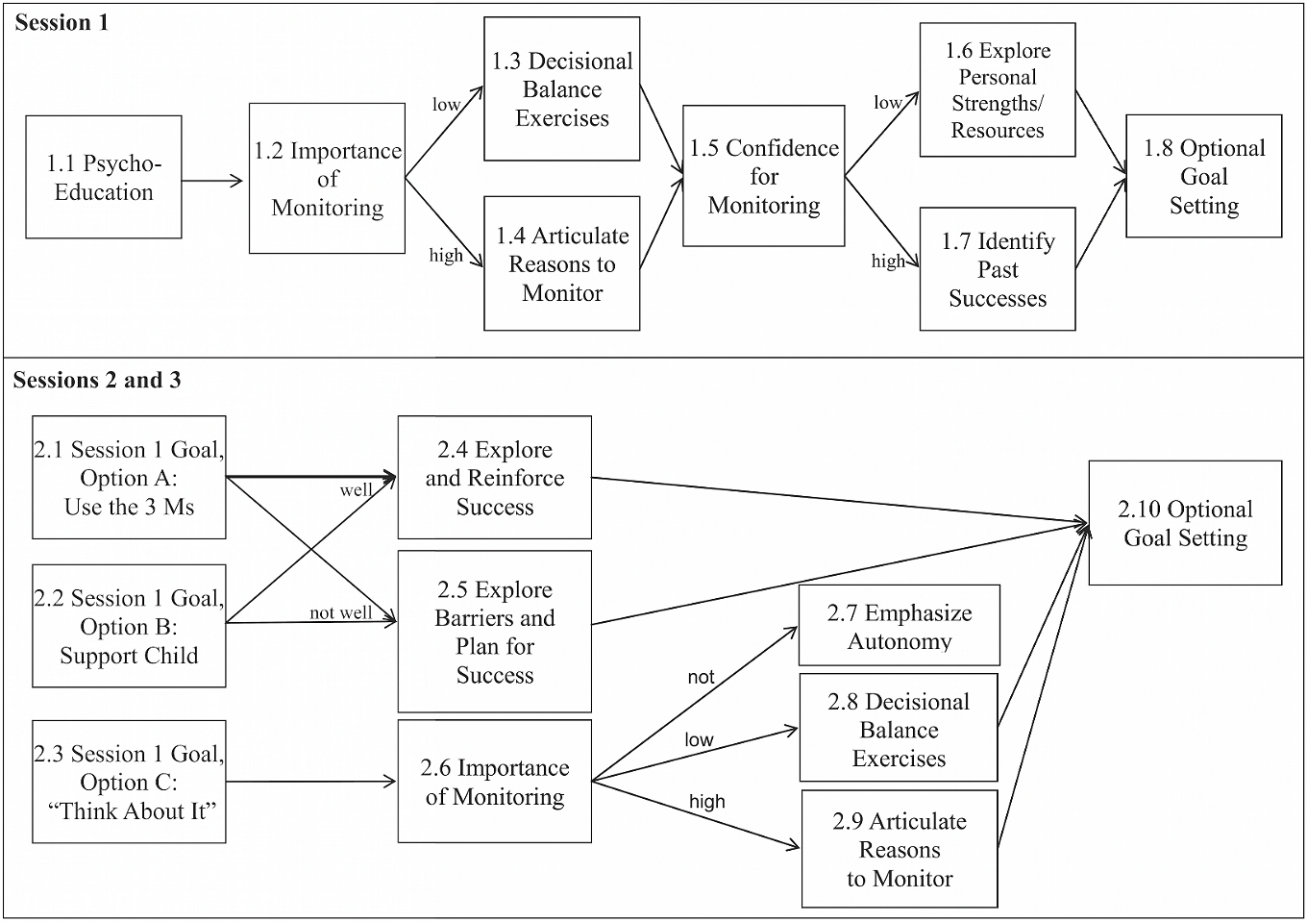
Flow chart of The 3 Ms Intervention.

The behavioral targets were based on three recommendations, which were termed The 3 Ms: (1) Watch your child give as many doses of insulin each day as possible (Medicine), (2) Check your child’s blood glucose meter at least once a day (Meter), and (3) Eat at least one meal each day with your child (Meals). The name The 3 Ms was chosen to function as a mnemonic to increase the likelihood that caregivers would recall these three key behaviors after the conclusion of the intervention. Figure 1 illustrates the various pathways through which participants could progress through the intervention.

To enhance caregivers’ knowledge of the importance of parental monitoring, an actor portraying a physician in a video clip delivered a small amount of psychoeducation at the beginning of Session 1 (Component 1.1). The goal of providing this information was to educate the participant about the recommended behavior change (ie, what parental monitoring is), its key features (ie, what behaviors constitute parental monitoring), and its benefits (ie, how daily parental monitoring of diabetes care is related to improved diabetes management and diabetes health). This psychoeducation was reinforced using a brief peer testimonial video clip in which an actor portraying a parent of a child with diabetes described her (fictional) experience with increasing parental monitoring of daily diabetes care. In this video clip, the parent describes how she came to learn of her child’s suboptimal diabetes care behavior and the resulting decline in his health status. She then recounts how increasing her parental monitoring led to improved diabetes care and several associated behavioral changes, eg, increased school performance. Providing such information is consistent with the IMB model, which suggests motivational approaches are most effective in the context of sensitively provided information about a health-related behavior [68]. In order to increase cultural competency, the scripts for both video clips were reviewed and tailored for appropriate language, communication style, and content by a pediatric health behavior researcher with expertise in developing interventions for urban, minority adolescents, specifically African American adolescents. In addition, the actors selected for the roles in the video clip were African American.

After educating participants about parental monitoring and its potential benefits, all participants were asked to rate the importance of implementing The 3 Ms using an adapted version of the Rollnick Readiness Ruler [69] (Component 1.2). Participants’ perceptions of the importance of implementing The 3 Ms determined which one of two distinct treatment components they received. Participants reporting low importance for parental monitoring were directed through exercises to explore their ambivalence and/or low motivation for increasing their parental monitoring behavior (Component 1.3). Participants reporting high importance for parental monitoring completed activities designed to reinforce their belief in the value of monitoring (Component 1.4).

Both groups were then directed to a ruler assessing their confidence in implementing The 3 Ms (Component 1.5). Those with low confidence were branched to activities designed to build confidence in implementing The 3 Ms (Component 1.6) and those with high confidence were reinforced (Component 1.7)*.* All participants ended the session with a goal-setting component where participants were given the option of choosing three goals: “use The 3 Ms”, “try other strategies to support their child’s diabetes care”, or “think about it” (Component 1.8). They recorded their goal on a goal-setting worksheet provided by study staff who made a single photocopy that was mailed to the participant approximately two months later. Session 1 required approximately 15-20 minutes to complete.

At their next diabetes clinic visit (approximately 3 to 4 months later), participants began Session 2 by selecting the parental monitoring goal they had worked on since Session 1. Participants who reported their goal was “use The 3 Ms” (Component 2.1) or “try other strategies to support their child’s diabetes care” (Component 2.2) were then asked to describe their experience implementing that goal. Those reporting positive progress toward their goal were directed to activities designed to reinforce their success and bolster their confidence (Component 2.4). Those less positive about their progress were branched to exercises designed to explore the barriers they might have encountered and bolster their confidence to continue trying to implement their goal (Component 2.5). Both groups ended the session with the goal-setting component (2.10).

Participants whose reported goal was to “think about” parental monitoring (Component 2.3) were directed to an importance reassessment (Component 2.6) to gauge their current readiness to change. Participants who indicated that parental monitoring was not important received an autonomy-supportive message acknowledging that the decision to monitor was their own and encouraging them to continue to think about it (Component 2.7). Those reporting low or high importance for monitoring were branched to motivation-enhancing exercises tailored to their specific level of readiness. Those with low motivation were directed to exercises designed to explore their ambivalence for increasing their parental monitoring behavior (Component 2.8), whereas those with high importance completed activities reinforcing their reasons for monitoring (Component 2.9). Both groups were then directed to the goal session component (2.10). Session 2 required approximately 12-15 minutes to complete.

Session 3 was a re-administration of Session 2 and was completed approximately 3 to 4 months after completing Session 2 (ie, at their next diabetes clinic visit). Participants again identified the parental monitoring goal they had been working on since their last session and proceeded through the intervention session as described above. Session 3 ended with a motivational message encouraging the caregiver to communicate with their health care team should they have questions in the future.

**Figure 2:**
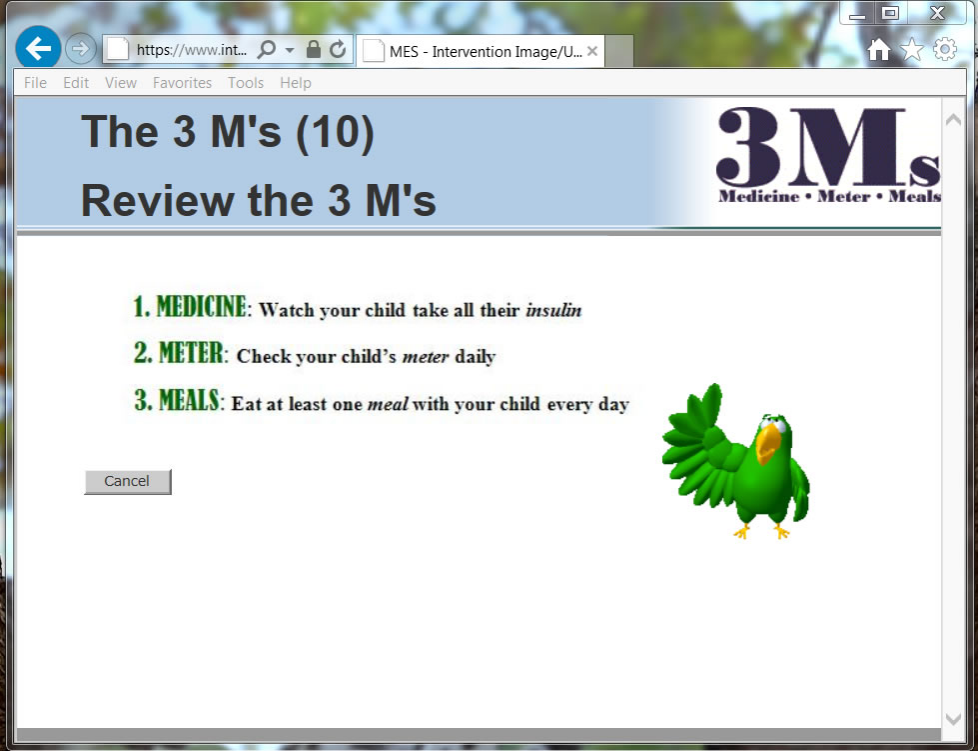
Screenshot of The 3 Ms Intervention.

Sessions were delivered by an animated character, or avatar, that has previously been used successfully (received high satisfaction ratings) with African American populations (see Figure 2)[55,56,70,71]. The avatar’s communication style and demeanor were consistent with principles of MI, suggesting that factors such as empathy, optimism, and congruence are strongly related to more client behavior change [72]. Previous research has demonstrated that computer avatars can successfully use these relational skills [73]. Significant efforts were made to ensure that the avatar delivered The 3 Ms with high MI fidelity. For example, throughout the intervention, the avatar reflected back the participant responses with affirmations to boost self-efficacy and statements emphasizing personal choice. As depicted in Figure 1, participants’ intervention trajectories were tailored based on their responses to importance and confidence assessments and the avatar’s communication was scripted within each trajectory to be consistent with the participants’ current readiness to change.

Once developed, the intervention was reviewed by two experts. A pediatric diabetologist (KM) reviewed recommendations in The 3 Ms for consistency with the treating health care center’s diabetes treatment guidelines as well as the recommendations of the American Diabetes Association [67]. CJ, a pediatric health behavior researcher with expertise in developing interventions for urban, minority adolescents, also reviewed the complete intervention to improve its cultural appropriateness. These reviews resulted in minor edits to the content and language of the sessions.

### Beta Test

The primary caregivers of five African American youth aged 10-13 years diagnosed with T1D for a minimum of one year (ie, the target population for the intervention) were recruited from a large, urban teaching hospital located in a large Midwestern city using convenience sampling procedures. Primary caregiver was defined as the person who lived with and helped the child with his/her diabetes care most of the time. Table 1 describes the sample characteristics. The diabetes clinic staff mailed letters of introduction to all eligible families. Disinterested families could opt-out of any further contact. Research staff followed up with the remaining families to assess their interest in participation. The research protocol was approved by the university’s Institutional Research Board. All caregivers provided informed consent to participate.

**Table 1:** Participant characteristics.

Participant number	Caregiver gender	Child age (years)	Child gender	Illness duration (years)
1	Female	12.4	Female	4.0
2	Female	12.2	Female	2.4
3	Female	11.5	Female	2.7
4	Female	11.6	Male	2.0
5	Female	11.0	Male	5.8

### Participants’ Ratings of The 3 Ms

Caregivers participated in one research visit at the research offices. During this visit, they first reviewed the intervention as if they were a participant, and then completed a semi-structured individual interview designed to elicit their feedback on the intervention’s appropriateness, utility, and cultural relevance. Interview questions were both closed-ended (“On a scale of 1 to 10, with 1 being not at all helpful and 10 being extremely helpful, how helpful do you think the computer program will be in helping the caregivers of children with diabetes identify ways to better supervise their child’s diabetes care?”) and open-ended (“If you could change any part of the program, what would you change?”). Caregivers received a US$25 gift card to a major retailer for completing the study.

### Thematic Analysis

In preparation for analysis, the interviews were transcribed by a professional transcription service. The transcribed interview data were analyzed using thematic analysis conducted in NVivo 9, a qualitative data analysis software package [74]. Responses to closed-ended questions were tallied (see Table 2). Two coders, one being the primary author and the second a research assistant, independently coded responses to the open-ended questions using the procedure outlined by Aronson [75] with additional guidance from Braun [76]. First, coders reviewed each transcript identifying responses to each interview question. These initial themes corresponded to the broad areas of interest the interview was designed to assess. They included participants’ perceptions of the helpfulness of the intervention, enjoyable intervention components, key intervention components, intervention acceptability, intervention’s impact on diabetes, and preference for motivational versus directive physician psychoeducation. Coders coded transcripts independently, then met to compare their coded transcripts before proceeding. Coding discrepancies were discussed and resolved; the consensus coded transcript was used for the subsequent coding pass. In the second coding pass, caregivers’ responses to each question were examined to identify commonalities, or themes. Using the caregivers’ own words, these themes were labeled, described, and applied to all the data, across all interview questions. Coders again coded independently, met to compare their coded transcripts, and reconciled coding discrepancies. Throughout this process, the theme descriptions were continuously augmented and clarified to ensure that all participants’ experiences were represented. The final result of this work was four themes, described more fully in the results below. Two themes described aspects of the structure and delivery of the intervention, the avatar, and The 3 Ms mnemonic that participants particularly enjoyed. Two themes pertained to the intervention’s relevance and utility, the importance of supervision, and shared experience.

**Table 2:** Caregiver ratings of The 3 Ms intervention.

Participant number	Helpfulness rating^a^	Recommendation rating^b^	Increased likelihood of talking to child about diabetes?	Physician video preference (Directive vs Motivational)
1	8	9	No	Directive
2	10	10	Yes	Motivational
3	9	10	Yes	Directive
4	10	10	Yes	Directive
5	10	10	No	Directive

^a^Anchored with 1 corresponding to “not at all helpful” and 10 to “extremely helpful”.

^b^Anchored with 1 corresponding to “not at all” and 10 is “definitely”.

## Results

### Participants’ Ratings of The 3 Ms

Table 2 presents caregivers’ responses to the closed-ended questions. All caregivers rated the helpfulness of The 3 Ms in helping caregivers identify ways to better supervise their child’s diabetes care at least an 8 out of 10, where 10 corresponded to a perception that the intervention was “extremely helpful”. Four caregivers rated their likelihood of recommending The 3 Ms program a 10, meaning that they would “definitely” recommend The 3 Ms to another parent of a child with diabetes. Three caregivers reported that The 3 Ms program changed the overall likelihood that they would talk to their child about diabetes, whereas two caregivers reported that they already engaged in regular conversations about diabetes care and related topics with their child.

### Thematic Analysis

Thematic analysis of caregivers’ responses to the open-ended questions identified two themes regarding the structure and delivery of the program. All five caregivers reported that the use of an animated avatar to deliver the intervention increased their enjoyment of the program: “The little avatar made it exciting, not seem so boring”; “That was cute. It made it interesting.” In addition, four of the five caregivers mentioned the intervention mnemonic (The 3 Ms) in their interviews. Caregivers found the mnemonic to be useful for remembering key monitoring behaviors, “something that you can remember”, and simple enough to be easily implemented, “The 3 Ms are something that you can keep up with.”

In addition, two broad themes related to the intervention’s relevance and utility were identified.

### Importance of Supervision

All the caregivers who reviewed the intervention found it to be a useful reminder regarding the importance of supervising their child’s diabetes care. Specifically, caregivers remarked that it was helpful to be reminded that even “good” children—those demonstrating independence and responsibility—need to be supervised. Caregivers perceived the intervention to be useful because parents often reduce their supervising behavior over time as they fall into routines, become lax or frustrated, or otherwise fatigued.

You do learn that, no matter how responsible you think your child is, they’re not as responsible as you really think they are, and you really do need to check and monitor behind them. Not because they’re bad children, just, you know, they’re children.

I’m always on my daughter about, you know, thinking that she’s doing it and then she’s not or she’s saying she is and, you know. Now I realize it is hard for them when they get to a certain stage or age to maintain. And I’m just taking for granted that she’s doing it.

So, I mean, it’s kind of a reminder and kind of a wakeup call in the same sense, you know. We still need to, even though they’re getting older, we still need to monitor what’s going on. Because we all get comfortable with thinking that we’re doing the right things and, even though we could be, but sometimes I’m sure we all fall short on checking the meter or not because the kid said, “I did it”.

### Shared Experience

All five caregivers reported the peer testimonial provided them with a feeling of having a shared experience. Specifically, they reported hearing the perspective of another parent who thought her child was independently and responsibly managing diabetes only to learn that the child was not doing as well as she thought was extremely helpful. This experience provided them with a sense that “I’m not alone” and that other parents of young adolescents with chronic illnesses struggle with these issues too.

Her daughter seemed similar to mine, you know. My child caught on very fast, way quicker than I did. Before she got released from the hospital, she knew how to give shots, blood sugar. The doctors and everyone made sure we were prepared when we got home. And so, her situation seemed similar that, because my child learned it so soon, I thought she was ready to take more responsibility towards her diabetes, and found out, like that mom, that my child wasn’t as responsible as I thought she was. So, it was helpful to see that somebody else was going, to hear, rather, and see that someone else was going through the same things. So, letting parents know you’re not alone out there. Your child is not the only one. There’s nothing wrong with your child.

While feedback was positive overall, caregivers did raise one concern with the intervention. Two caregivers suggested that The 3 Ms might be most appropriate for caregivers of newly diagnosed children. Specifically, they indicated that “after so many years, you’ve heard all of this from the doctors”. One of the two did acquiesce that “it’s still helpful to hear some of the things Dr. Moore (the actor portraying a physician in the video) said”. A third caregiver suggested that The 3 Ms was a useful “refresher” after having been diagnosed for many years:

I think it’s pretty good though because the only other program or training or what to do type of thing was at the beginning when she was diagnosed, when they give the classes and things like that. So, I mean, coming back years later, and even though you have your hospital visit and clinic visits and stuff like that, it’s still different to be able to, I guess a refresher type thing.

Finally, caregivers were also asked to review two versions of the physician psychoeducation video clip. In one video, the physician’s speech was scripted to be consistent with the principles of MI. To illustrate, autonomous decision making was emphasized at several points. For example, in her initial comments, the physician acknowledges that “How you parent your teen with diabetes is up to you and your family” and “you can decide for yourself just how important it is to be involved in your teen’s diabetes care as your teen gets older”. Later, when making her recommendation, she encouraged parents to monitor daily, but explained that it was their choice whether they changed their monitoring behavior, “So, those are the facts. What you do with them is up to you.” Also consistent with MI, before providing the caregiver with information about parental monitoring, the physician asks permission to give that information, albeit indirectly, “I hope you won’t mind if I take just a minute to tell you a little about parenting a teen with diabetes”.

In contrast, the other physician video was more directive; in this version, she stated clearly and firmly that that it was in a parent’s best interests to monitor their child’s diabetes care daily: “As a doctor, I must tell you that it is very important for you to supervise your child’s diabetes care every day”. Her initial comments and recommendations for monitoring were similarly presented in a very direct manner emphasizing the physician’s expertise and authority in this area, “As a doctor, I must tell you that it is very important for you to supervise you child’s diabetes care every day” and “the best advice I can give you is to start daily supervision as soon as possible”. This approach is consistent with the “Advise to Quit” recommendation of the “5 As” smoking cessation intervention, a brief clinical intervention grounded in empirical support and expert opinion that states, “In a clear, strong, and personalized manner, urge every tobacco user to quit” [77].

The videos were presented in alternating order to consecutive participants. Four caregivers preferred the directive video over the motivational video. When asked to explain their preference, caregivers stated that the directive video conveyed a greater sense that parental monitoring was important and contained more information (despite both videos containing exactly the same informational content). Caregivers also indicated a directive approach was appropriate for discussions of children’s health: “I think sometimes you just don’t need to always sugarcoat things. You just need to tell it how it is, because it’s a very serious disease and you can die from it.”

## Discussion

### Principal Findings

The results of this study suggest that The 3 Ms is an appropriate and acceptable intervention for caregivers of children with T1D. Caregivers provided positive feedback via both quantitative ratings and qualitative comments. Caregiver comments suggested that the intervention was both helpful and enjoyable.

This study provided insight into caregivers’ preferences for receiving psychoeducation related to their children’s health delivered by a computer-based intervention using a motivation-enhancing framework. Although MI theory suggests that behavior change information is best received under conditions where individuals’ autonomous decision making is supported [66], four of the five caregivers in this study preferred a directive approach over a motivation-enhancing approach for the information about the importance of parental monitoring presented in video clips. One interpretation of this finding, supported by caregivers’ comments, is that motivational approaches may be preferred when discussing one’s own behavior or health, but when discussing a child’s health, caregivers prefer a directive approach. The participants in this study preferred a directive approach, a preference that will be honored in the pilot study; however, more research is needed to empirically test whether a directive approach is more effective at evoking behavior change than motivational approaches in this context.

To increase the cultural competency of the intervention, a deliberate effort was made to select actors to portray the physician in the psychoeducational video clip and the caregiver in the peer testimonial with whom the target population would identify, that is, African American actors. Although participants were not directly asked about their ability to relate to the physician and caregiver portrayed in the video clips, a primary theme, Shared Experience, emerged from the qualitative analysis indicating that video clips did evoke such feelings. A meta-analysis examining the effect of culturally adapted interventions (interventions designed for a specific cultural group) found that culturally tailored intervention were four times more effective than interventions that were not culturally tailored [78]. Additional research is needed to form a conclusion about the effect of the cultural tailoring of The 3 Ms intervention on participant outcomes.

### Limitations

This study is limited by a small sample size. However, the sample size is justified by a need for small scale intervention development studies to develop and preliminarily validate interventions prior to resource-intensive randomized controlled trials [79]. A second limitation is the feasibility and acceptability of integrating a computerized intervention into routine diabetes clinical practice. Although at least two previous studies have reported successful testing of computer-delivered interventions into outpatient [55] and inpatient medical settings [80], studies examining the implementation of a such an intervention using typical clinical support staff (ie, not research staff) is needed.

### Future Steps

The next step of this research, currently under way, is a pilot randomized controlled trial to examine the ability of The 3 Ms to influence health outcomes in this high risk population. The pilot study is utilizing a randomized, repeated measures design where participants are allocated to one of three intervention arms. In Arm 1, 30 caregivers will receive the motivation-enhancing intervention targeting parental monitoring of their child’s daily diabetes care described here and their children will receive a similar intervention targeting children’s own daily diabetes care. In Arm 2, 30 caregivers will receive the motivation-enhancing intervention but their children will receive an attention-control intervention, three computer-delivered sessions of similar duration that provide diabetes education unrelated to parental monitoring or daily diabetes care. The educational topics are diabetes-related emergency preparedness, traveling with diabetes, and smoking and diabetes. In Arm 3, 30 caregivers and their children will both receive the control intervention.

Pilot study participants will be recruited from an urban, Midwestern medical center serving a patient population with a significant representation of ethnic minorities and families of lower socioeconomic status. Participants will complete four study visits; the first three will include both study-related assessments as well as the delivery of the interventions and the fourth will be a follow-up assessment only. The primary outcomes will be caregiver motivation for parental monitoring of daily diabetes care and adherence to the diabetes care regimen. Secondary outcomes will include parental monitoring behavior and glycemic control (hemoglobin A1c). At the writing of this report, recruitment into the pilot study was well under way; 67 participants were enrolled and randomized. Thus far, 91% of participants have been successfully retained across intervention arms.

## Abbreviations

IMB: Information-Motivation-Behavioral
MI: motivational interviewing
T1D: type 1 diabetes
